# Canonical TGF-β signaling regulates the relationship between prenatal maternal depression and amygdala development in early life

**DOI:** 10.1038/s41398-021-01292-z

**Published:** 2021-03-15

**Authors:** Anqi Qiu, Han Zhang, Changqing Wang, Yap-Seng Chong, Lynette P. Shek, Peter D. Gluckman, Michael J. Meaney, Marielle V. Fortier, Yonghui Wu

**Affiliations:** 1 Department of Biomedical Engineering, National University of Singapore, Singapore, Singapore; 2 The N.1 Institute for Health, National University of Singapore, Singapore, Singapore; 3 Smart Systems Institute, National University of Singapore, Singapore, Singapore; 4 Department of Biomedical Engineering, Johns Hopkins University, Baltimore, MD USA; 5 Singapore Institute for Clinical Sciences, Singapore, Singapore; 6 Department of Obstetrics & Gynaecology, Yong Loo Lin School of Medicine, National University of Singapore, National University Health System, Singapore, Singapore; 7 Department of Pediatrics, Khoo Teck Puat – National University Children’s Medical Institute, National University of Singapore, Singapore, Singapore; 8 Ludmer Centre for Neuroinformatics and Mental Health, Department of Psychiatry, Faculty of Medicine, McGill University, Montreal, QC Canada; 9 Department of Diagnostic and Interventional Imaging, KK Women’s and Children’s Hospital, Singapore, Singapore

**Keywords:** Molecular neuroscience, Depression

## Abstract

Canonical transforming growth factor-beta (TGF-β) signaling exerts neuroprotection and influences memory formation and synaptic plasticity. It has been considered as a new target for the prevention and treatment of depression. This study aimed to examine its modulatory role in linking prenatal maternal depressive symptoms and the amygdala volumes from birth to 6 years of age. We included mother–child dyads (birth: *n* = 161; 4.5 years: *n* = 131; 6 years: *n* = 162) and acquired structural brain images of children at these three time points. Perinatal maternal depressive symptoms were assessed using the Edinburgh Postnatal Depression Scale (EPDS) questionnaire to mothers at 26 weeks of pregnancy and 3 months postpartum. Our findings showed that the genetic variants of TGF-β type I transmembrane receptor (TGF-βRI) modulated the association between prenatal maternal depressive symptoms and the amygdala volume consistently from birth to 6 years of age despite a trend of significance at 4.5 years of age. Children with a lower gene expression score (GES) of TGF-βRI exhibited larger amygdala volumes in relation to greater prenatal maternal depressive symptoms. Moreover, children with a lower GES of the TGF-β type II transmembrane receptor (TGF-βRII), Smad4, and Smad7 showed larger amygdala volumes at 6 years of age in relation to greater prenatal maternal depressive symptoms. These findings support the involvement of the canonical TGF-β signaling pathway in the brain development of children in the context of in utero maternal environment. Such involvement is age-dependent.

## Introduction

Intrauterine exposure to maternal depression has long-term impacts on emotional^1^, behavioral^2^, and cognitive outcomes^3^ and increases the later risk for depression in the offspring^4^. Increasing evidence also suggests that maternal depression during pregnancy influences the brain development of offspring^5–8^. Children born to mothers with prenatal depression show alterations in the microstructure and functional connectivity of the amygdala at birth^9,10^, greater functional connectivity of the amygdala with cortical regions involved in emotion regulation in 6-month-old infants^6^ and 4-year-old children^5^, a larger right amygdala volume in young girls at age of 4 and 7 years^7,11^, and alterations of the amygdala–prefrontal structural circuit from birth to early childhood^12^. These findings suggest that prenatal maternal depression has a potential long-term impact on the brain development of offspring, particularly in the emotion-related brain regions.

Biological mechanisms for linking the relationship of pregnancy depression and child brain development are not fully understood. Convergent evidence from neuroimaging research suggests that maternal–placental–fetal stress and inflammation are likely biological transmission pathways^11,13–15^. A higher maternal cortisol level during pregnancy predicts greater amygdala functional connectivity with the supramarginal gyrus in female neonates^16^ and a larger amygdala volume in 7-year-old girls^11^. Moreover, depressive symptoms are associated with elevated serum proinflammatory cytokines in pregnant women^17^. Maternal interleukin 6 (IL-6) can cross the placenta^18^, stimulate prenatal cytokine production^19,20^, and alter inflammatory signaling in the fetal environment^20^, which may have a significant impact on fetal brain development. A higher maternal IL-6 level is associated with rapid amygdala growth from 4 to 36 months in macaque offspring^21^. In humans, elevated maternal IL-6 during pregnancy is related to a larger amygdala volume^13^, structural and functional amygdala connectivity^22^, and specific brain networks in neonates^14^. These findings provide new evidence in macaque and humans linking maternal depression and inflammation during pregnancy with offspring brain development. Nevertheless, there is a lack of knowledge on how inflammatory genetic variants of the offspring modulate such a link for a better understanding of the susceptibility of offspring brain development to intrauterine conditions.

**Fig. 1:**
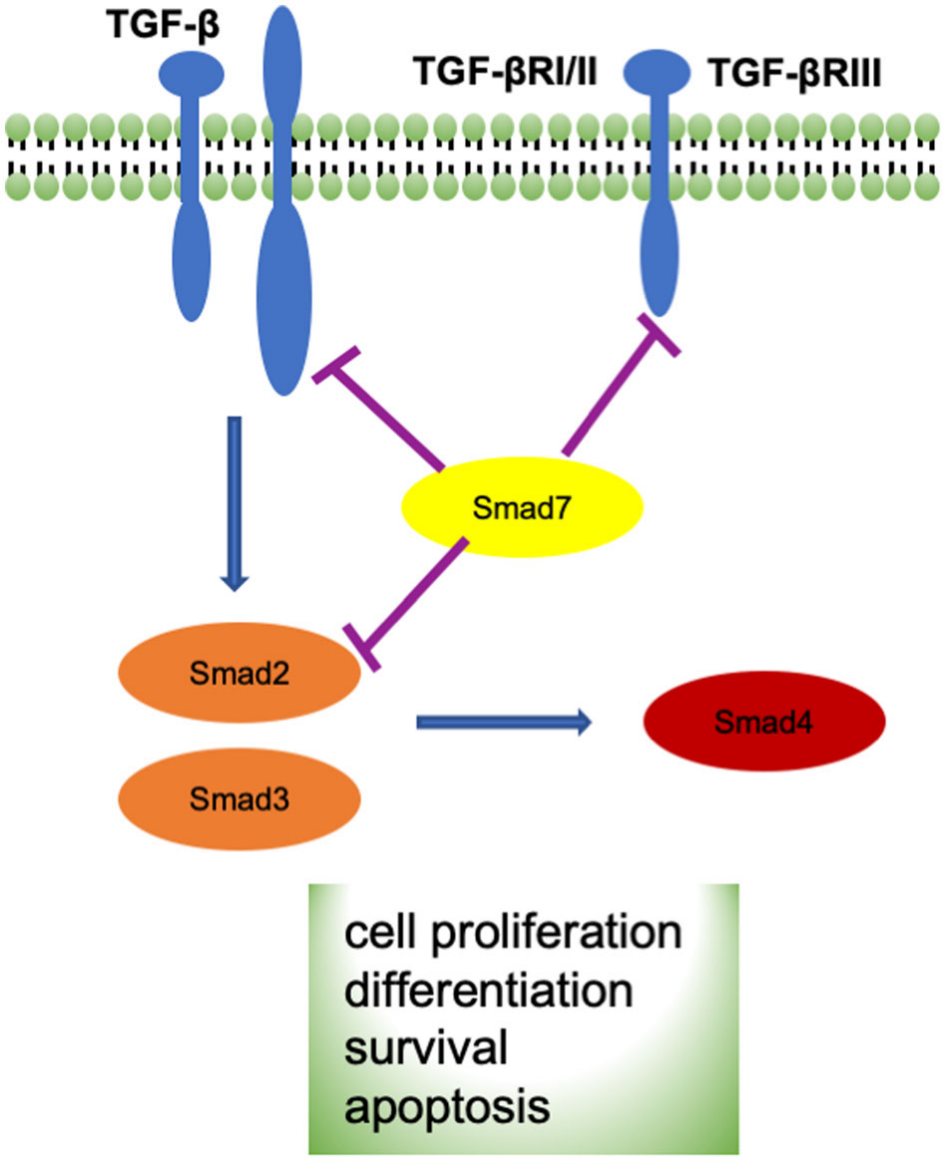
Canonical TGF-β signaling pathway. Canonical transforming growth factor beta (TGF-β) signaling is a process that involves ligands (TGF-β1, TGF-β2, TGF-β3), receptors (TGFBRI/II/III), receptor-activated SMADs (R-Smads: Smad2/3; I-Smads: Smad7), and the common Smad (co-Smad: Smad4). TGF-β superfamily ligands bind to a type II receptor, which recruits and phosphorylates a type I receptor. The type I receptor then phosphorylates R-Smads that can bind Smad4. R-Smads/co-Smad complexes accumulate in the nucleus where they act as transcription factors and participate in the regulation of target gene expression.

Canonical transforming growth factor-beta (TGF-β) signaling (Fig. 1) is an anti-inflammatory signal that exerts neuroprotective effects and influences memory formation and synaptic plasticity and has recently received attention in depression research. Prenatal stress-exposed animals exhibit reduced protein levels of the major ligands of the canonical TGF-β pathway, TGF-β1–3, in the brain^23,24^. Antidepressant drugs, such as tianeptine and venlafaxine, can reverse these effects^23^. The canonical TGF-β signaling cascade initiates TGF-β binding to the type II transmembrane receptor serine/threonine kinases (TGF-βRII) that phosphorylate and activate the type I receptor (TGF-βRI). Activated TGF-βRI can phosphorylate the downstream effectors, Smad2 and Smad3, that associate with Smad4 and Smad7^25^. In animals, prenatal stress is associated with the reduced levels of TGF-βRI and TGF-βRII and alters the level of Smad7 in the frontal cortex^23^. Rats with chronic mild stress exhibit significant decreases in TGF-β in the brain^26^. Similar to the findings in animals, patients with depression show a reduction in TGF-β serum levels^27,28^ that correlate with depression severity^29^ and significantly contribute to treatment resistance in major depressive disorder (MDD)^30^. Moreover, TGF-β1 has no effect on the basal release of corticotropin-releasing factor (CRF) but selectively blocks the acetylcholine-induced release and inhibits arginine vasopressin (AVP) in both the amygdala and hypothalamus^31^, suggesting that TGF-β1 may modulate the hypothalamic–pituitary–adrenal axis activation by antagonizing (acetylcholine-evoked) CRF and AVP release. These findings support a role for the amygdala in the communication between the neuroendocrine and immune systems^31^.

This study aimed to answer two questions: (1) does the canonical TGF-β signaling pathway play a role in modulating the link between pregnancy depression and child amygdala development in early childhood? (2) Is there any specific timing (e.g., in utero or early childhood) when the canonical TGF-β signaling pathway plays such a role? We investigated these questions using the dataset from a prospective population-based cohort study, Growing Up in Singapore Towards healthy Outcomes (GUSTO), including maternal mental health, child genotype, and brain images at birth, 4.5, and 6 years of age. Due to the child compliance in the magnetic resonance imaging (MRI) scanner, GUSTO only acquired MRI brain data at 4.5 years of age after the neonatal scan. We employed an advanced gene set-based mixed effect model for gene–environment interaction (MixGE)^32^ to examine interactive effects between child genetic variants of each gene in the canonical TGF-β signaling pathway (Fig. 1) and prenatal maternal depressive symptoms on the amygdala volume at birth, 4.5, and 6 years of age. The MixGE took a set of single-nucleotide polymorphisms (SNPs) and examined not only accumulative but also heterogeneous interactive effects of these individual SNPs with prenatal maternal depressive symptoms on the amygdala volume at each time point. We expected that, in children with genetic variants related to the lower expression level of the TGF-β ligands and receptors, greater prenatal maternal depressive symptoms would correlate with a larger amygdala volume at a specific age. Our findings provided novel evidence that supports the canonical TGF-β signaling pathway as a biological mechanism linking pregnancy depression and brain development of the offspring.

## Materials and methods

### Subjects

The National Healthcare Group Domain Specific Review Board and the Sing Health Centralized Institutional Review Board approved the GUSTO cohort study. Mothers provided written consent. When children reached 6 years of age, oral consent was obtained from children.

Mother–child dyads who participated in this imaging genetic study were part of the GUSTO cohort whose study design and protocol were described in ref. ^33^. The GUSTO is a population-based birth cohort study. In the first-trimester clinic at the National University Hospital and KK Women’s and Children’s Hospital^34^ in Singapore, pregnant Asian women were screened. Pregnant women who were Singapore citizens or Permanent Residents of Chinese, Malay, or Indian ethnic background and had no prior history of mental illness, diabetes, other major illnesses, and substance use were recruited. Socioeconomic status (SES)^35^ represented by maternal education, ethnicity, and age were extracted from survey questionnaires conducted as part of a scheduled appointment during the 26th week of pregnancy. Birth outcomes, including gestational age, birth weight, Appearance, Pulse, Grimace, Activity, and Respiration (APGAR) score, and sex, were obtained from the hospital record.

**Fig. 2:**
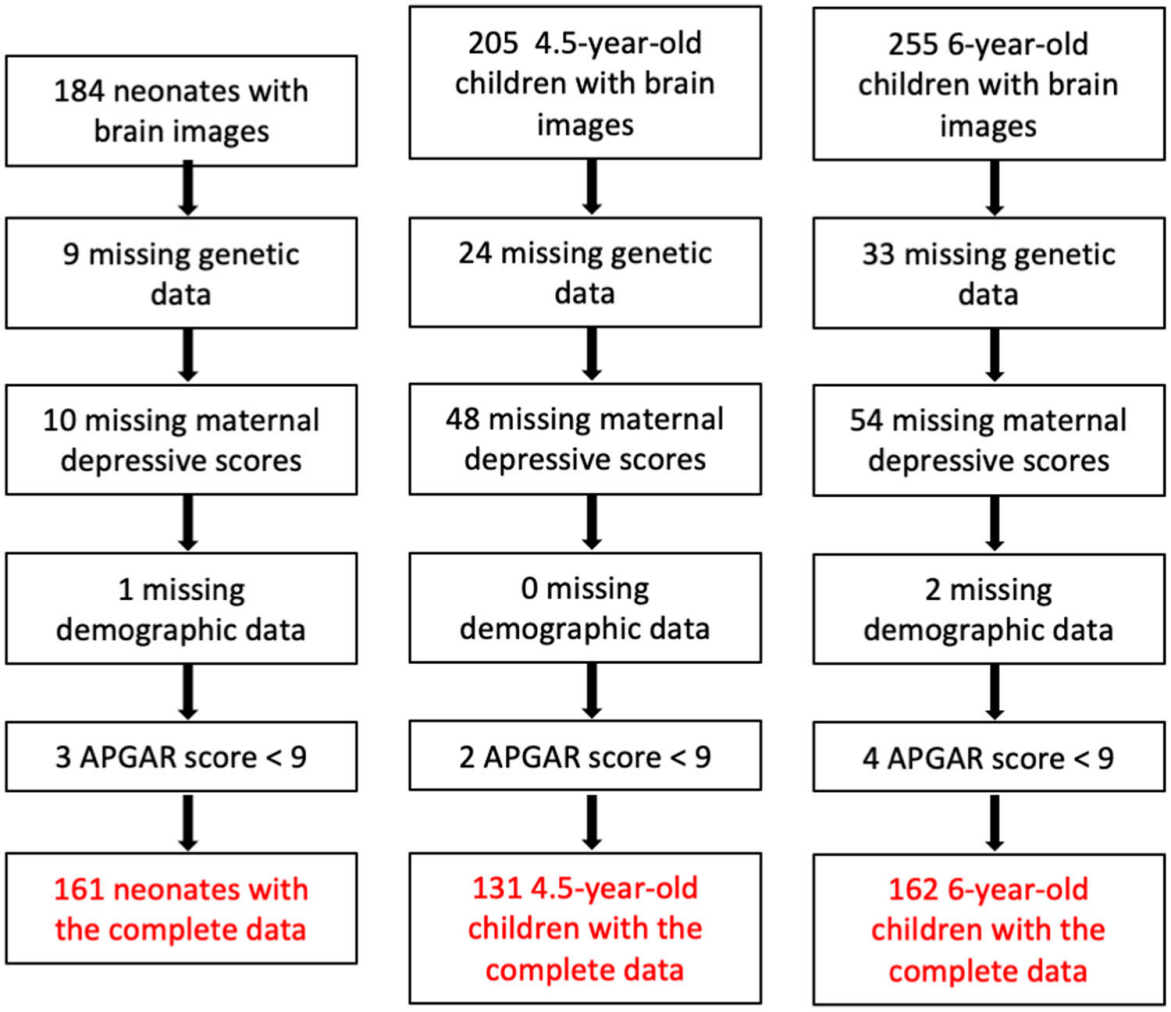
Subject selection. Flow chart of the subject selection.

This imaging study included children with maternal depression scales and no pregnancy complications and with gestational age, ≥34 weeks, birth weight ≥2 kg, and a 5-min APGAR score ≥9 to avoid potential effects of pregnancy and birth complications on brain development. Table 1 lists the demographic information used in this study. Figure 2 illustrates the flow chart of the subject exclusion based on the above criteria, resulting in 161 neonates, 131 4.5-year-old children, and 162 6-year-old children in this study.

**Table 1:** Demographics.

Measure	Neonatal sample	4.5-year-old sample	6-year-old sample
	(*n* = 161)	(*n* = 131)	(*n* = 162)
*Child characteristics*			
Gestational age (weeks), mean (SD)	38.9 (1.1)	38.9 (1.2)	39.1 (1.1)
Birth weight (g), mean (SD)	3087.1 (372.1)	3088.6 (393.3)	3110.0 (415.0)
APGAR score	≥9	≥9	≥9
Sex, male/female	87/74	57/74	70/92
Age (years), mean (SD)	0.027 (0.01)	4.58 (0.07)	6.05 (0.12)
*Mother characteristics*			
Prenatal maternal depression score, mean (SD)^a^	8.56 (4.50)	7.56 (4.66)	7.26 (4.22)
3-month postnatal depression score, mean (SD)	—	6.23 (4.80)	6.31 (4.78)
Maternal ethnicity, %			
Chinese	45.34	48.86	51.24
Malay	41.62	35.11	35.18
Indian	13.04	16.03	13.58
Maternal education, %			
Primary school	3.73	5.344	3.09
Secondary school	33.54	32.061	32.71
Pre-university, diploma or technical course	44.71	40.458	33.33
University undergraduate level	14.29	19.084	28.40
Above university undergraduate level	3.73	3.053	2.47

^a^Denotes statistical significance of the group difference.

### Maternal depressive symptoms

The Edinburgh Postnatal Depression Scale (EPDS) questionnaire^36^ was administered to mothers at 26 weeks of pregnancy and 3 months postpartum to quantify perinatal depressive symptomatology. The EPDS is a widely used 10-item self-report scale designed as a screening instrument for postnatal depression, but it is also valid for use during pregnancy. Each item of the EPDS is scored on a 4-point scale (0–3), for a total score of 30. A higher total score indicates the presence of more depressive symptoms.

Beck’s Depression Inventory-II (BDI-II) was administered to mothers at 1, 2, 3, 4, and 6 years postpartum. The BDI-II is a widely used 21-item questionnaire that assesses the existence and severity of symptoms of depression and predicts the severity of clinical depressive symptoms^37^. Each item of the BDI-II is scored on a 4-point scale (0–3). Higher total scores indicate more severe depressive symptoms.

Prorating imputation was performed when 8 or 9 questions were answered on the EPDS or 19 or 20 questions were answered for the BDI-II. The BDI scores were highly correlated with the EPDS score at 3 months postpartum (*r* > 0.5, *p* < 0.001). Longitudinal trajectory analysis on postnatal maternal depressive symptoms from 3 months to 6 years postpartum showed their stability across time as illustrated in ref. ^5^. Hence, this study employed the EPDS score at 3 months postpartum to quantify the severity of postnatal maternal depressive symptoms and was used as a confounding variable in this study.

### MRI acquisition and analysis

#### Neonatal brain

Neonates at 5–14 days of age underwent axial fast spin-echo T_2_-weighted MRI scanning (repetition time (TR) = 3500 ms; echo time (TE) = 110 ms; field of view = 256 mm × 256 mm; matrix size = 256 × 256; 50 axial slices with 2.0 mm thickness) using a 1.5-T GE scanner at the Department of Diagnostic and Interventional Imaging of the KK Women’s and Children’s Hospital. Detailed acquisition and image quality check procedures were previously reported^38,39^.

Within individual subjects, a Markov random field model was used to automatically delineate the amygdala from the neonatal T2-weighted MRI data. The segmentation accuracy rate of the automatic segmentation against the manual segmentation was reported in previous publications^9,38,39^.

#### Child brain

GUSTO children underwent MRI scans at age of 4.5 years (4.44–4.79 years) and 6 years (5.83–6.61 years) using a 3-T Siemens Skyra scanner with a 32-channel head coil at KK Women’s and Children’s hospital. High-resolution isotropic T_1_-weighted magnetization prepared rapid gradient recalled echo was acquired with the protocol (TR = 2000ms, TE = 2.08 ms, field of view = 192 × 192 × 192 mm, matrix size = 192 × 192 × 192, inversion time = 877 ms, flip angle = 9°, scanning time = 3.5 min). Children went through an MRI home training program before the MRI visit and on-site MRI training and the image quality check was detailed in ref. ^7^. The amygdala was segmented from T_1_-weighted MRI using FreeSurfer^40^. Post-processing quality check was conducted following the instruction on https://surfer.nmr.mgh.harvard.edu/fswiki/FsTutorial/TroubleshootingData.

### SNP genotyping

The GUSTO study extracted genomic DNA from frozen umbilical cord specimens for children and from blood for mothers according to standard procedures. The samples were then genotyped on both Illumina OmniExpress and Exome arrays. Data were processed in GenomeStudio Genotyping Module™. Genotyping calls were made by the GenCall software that provides the GenCall score of each SNP probe and the call rate of each sample to rank and filter failed genotypes^41^. The genotypes with a GenCall score of <0.15 were not assigned genotypes^41^.

This study employed IMPUTE2 to impute the genotype data based on the reference of 1000 Genomes^42^. The locus range of each gene was first determined based on the hg19 database (https://genome.ucsc.edu). The SNPs in the locus range of each gene (±1 kb) were extracted from the imputed data and were then selected for this study when they did not deviate from Hardy–Weinberg proportions after correction for multiple comparisons. Table 2 lists the number of SNPs of each gene included in this study for children and mothers.

**Table 2:** Statistical *p* values for the interactions between the genetic variants and prenatal maternal depressive symptoms on the amygdala volumes at birth and at age of 4.5 and 6 years.

Gene	Number of SNPs	Birth		4.5 years		6 years	
		Left	Right	Left	Right	Left	Right
*Ligands*							
TGFβ1	66	0.455	0.451	0.681	0.955	0.831	0.652
TGFβ2	341	0.594	0.293	0.247	0.185	0.926	0.591
TGFβ3	125	0.447	0.304	0.171	0.271	*0.096*	0.132
*Type I and II receptors*							
TGFβ-RI	218	0.166	**0.004**	*0.058*	*0.054*	**0.043**	**0.037**
TGFβ-RII	435	0.373	0.722	0.623	0.815	**0.038**	0.605
TGFβ-RIII	1002	0.649	0.178	0.779	0.557	**0.014**	0.181
*R-Smads*							
Smad2	470	0.589	0.134	0.469	0.404	*0.074*	0.632
Smad3	611	0.161	0.379	0.421	0.432	0.253	0.978
*Co-Smads*							
Smad4	155	0.725	0.080	*0.800*	0.211	**0.014**	**0.007**
*I-Smads*							
Smad7	161	0.170	0.153	0.752	0.626	**0.031**	0.210

Bold: uncorrected *p* < 0.05; italics: uncorrected *p* < 0.1.

### Statistical analysis

We examined interactive effects between genetic variants of the TGF-β genes and prenatal maternal depressive symptoms on the amygdala volume at birth, 4.5, and 6 years of age using a gene set-based MixGE (https://bieqa.github.io/imaginggenetics.html)^32,43^. The MixGE model was in the form of

$${\mathrm{Y}} = Z\beta + {\mathrm{diag}}\left( E \right)G\pi + {\mathrm{diag}}\left( E \right)G\delta ,$$

where $Z = [X,E,G]$, where $X$ consisted of sex, age on the MRI visit day, SES, and ethnicity as covariates. For the time points of 4.5 and 6 years, the score of postnatal maternal depressive symptoms was also entered in *X* as a covariate. Here, we used maternal education, instead of household income, to represent SES since it was highly correlated with household income and a stable measure for SES across time. $Y$ was the amygdala volume. *E* was the score of prenatal maternal depressive symptoms, and *G* represented the SNPs of individual genes. ${\mathrm{diag}}\left( E \right)G$ therefore represented gene–environment interaction (G×E). $\pi$ was a fixed effect of G×E. $\delta$ were random effects of G×E. $\pi$ captured the accumulative G×E effects, whereas $\delta$ captured the heterogeneous G×E effects among all the SNPs. The same statistical analysis was applied to the amygdala volumes at each time point. Additional analysis was conducted when the variation of the same genes from mothers was also entered as additional covariate.

When the interaction was significant, we followed post hoc analysis where a genetic expression score (GES) was calculated for individuals by summing the number of alleles across the SNPs of the gene that was correlated with its expression level according to the existing expression quantitative trait loci (eQTL) database (https://gtexportal.org/). Here, this study employed the eQTL information to interpret the GES scale such that a higher GES indicates the genetic variation associated with a higher expression level of the gene. Simple slope analysis was then used to examine the associations between prenatal maternal depressive symptoms and the amygdala volume in the high GES group (+1 SD above the mean of GES) and low GES group (−1 SD below the mean of GES). The same set of covariates, such as sex, age on the MRI visit day, SES, ethnicity, and postnatal maternal depressive symptoms if it was 4.5 or 6 years of age were included in the post hoc analysis.

## Results

### Demographics

Table 1 lists the demographic information of children at birth and age of 4.5 and 6.0 years. There were no differences in gestational age, birth weight, APGAR, sex, maternal ethnicity, and education, as well as postnatal maternal depressive symptoms among the three age groups (*p* > 0.10). However, prenatal maternal depressive symptoms showed a statistically significant difference among the three age groups (*F*_2,451_ = 3.73, *p* = 0.025).

### Modulatory roles of TGF-β genetic variants in the relationship between prenatal maternal depressive symptoms and the amygdala volume

**Fig. 3:**
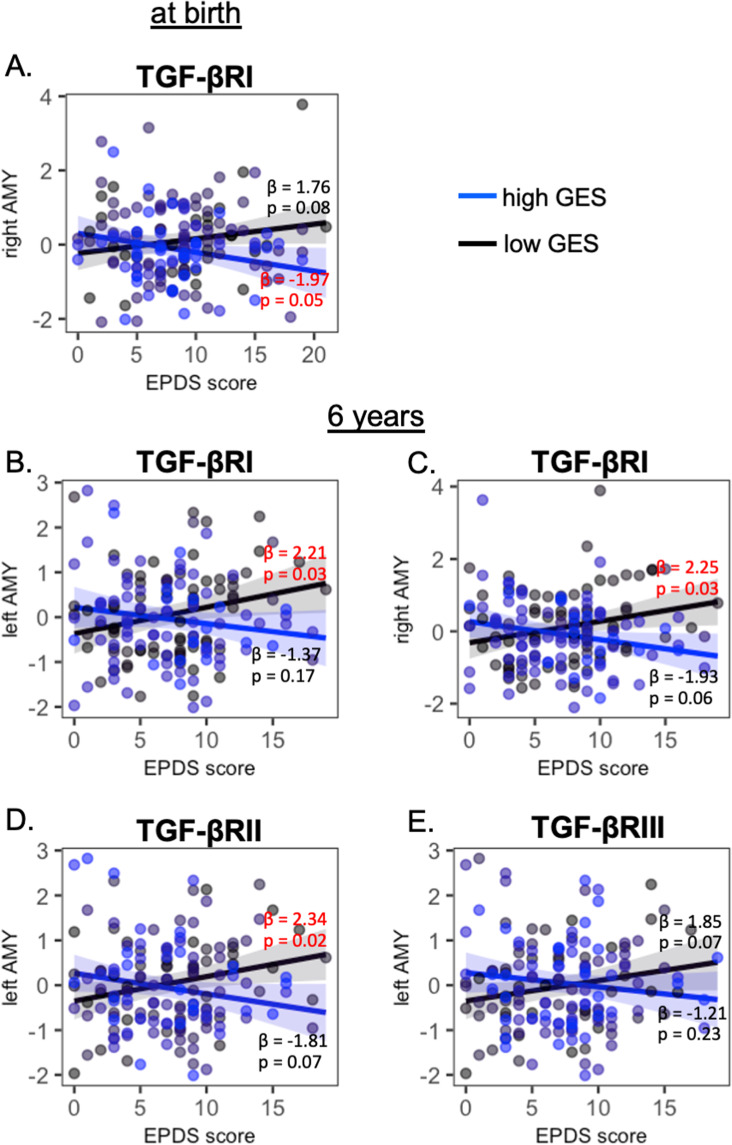
Interaction of prenatal maternal depression and TGF-β receptors on amygdala volumes. Post hoc analysis of the interaction between prenatal maternal depression and the genetic variants of TGF-βRI, TGF-βRII, and TGF-βRIII on the amygdala volume at birth (**A**) and at the age of 6 years (**B**–**E**). The black and blue lines/dots represent the groups with low and high genetic expression scores (GES), respectively. *β* and *p* values in red indicate statistical significance.

Supplementary Fig. S1 shows the gene expression levels in the canonical TGF-β signaling pathway in the first 8 years of life that were extracted from the BrainSpan sample (https://www.brainspan.org/static/download.html). Most of these genes showed upregulation in the first 4 years of life. We did not find significant interactions of prenatal maternal depressive symptoms with the genetic variants of TGF-β1–3, as well as Smad2 and Smad3, on the amygdala volume at any time point (Table 2), when sex, age on the MRI visit day, SES, and ethnicity were entered as covariates. Table 2 reports the uncorrected *p* values. TGF-βRI variants interacted with prenatal maternal depressive symptoms on the neonatal right amygdala volume (*p* = 0.004) and bilateral amygdala volumes at the age of 6 years (left: *p* = 0.043; right: *p* = 0.037). Only a trend of significance was observed in the bilateral amygdala volumes at the age of 4.5 years (left: *p* = 0.058; right: *p* = 0.054). Post hoc analysis revealed that, in neonates with a high GES of TGF-βRI, higher levels of prenatal maternal depressive symptoms were associated with a smaller right amygdala volume (*p* = 0.05; Fig. 3A). In children with a low GES of TGF-βRI, greater prenatal maternal depressive symptoms predicted greater left (*p* = 0.03; Fig. 3B) and right amygdala volumes at 6 years of age (*p* = 0.03; Fig. 3C).

Likewise, TGF-βRII and TGF-βRIII variants interacted with prenatal maternal depressive symptoms on the left amygdala volume at the age of 6 years (TGF-βRII: *p* = 0.038; TGF-βRIII: *p* = 0.014; Table 2). In 6-year-old children with a low GES of TGF-βRII, higher prenatal maternal depressive symptoms were related to a greater left amygdala volume (*p* = 0.02; Fig. 3D).

**Fig. 4:**
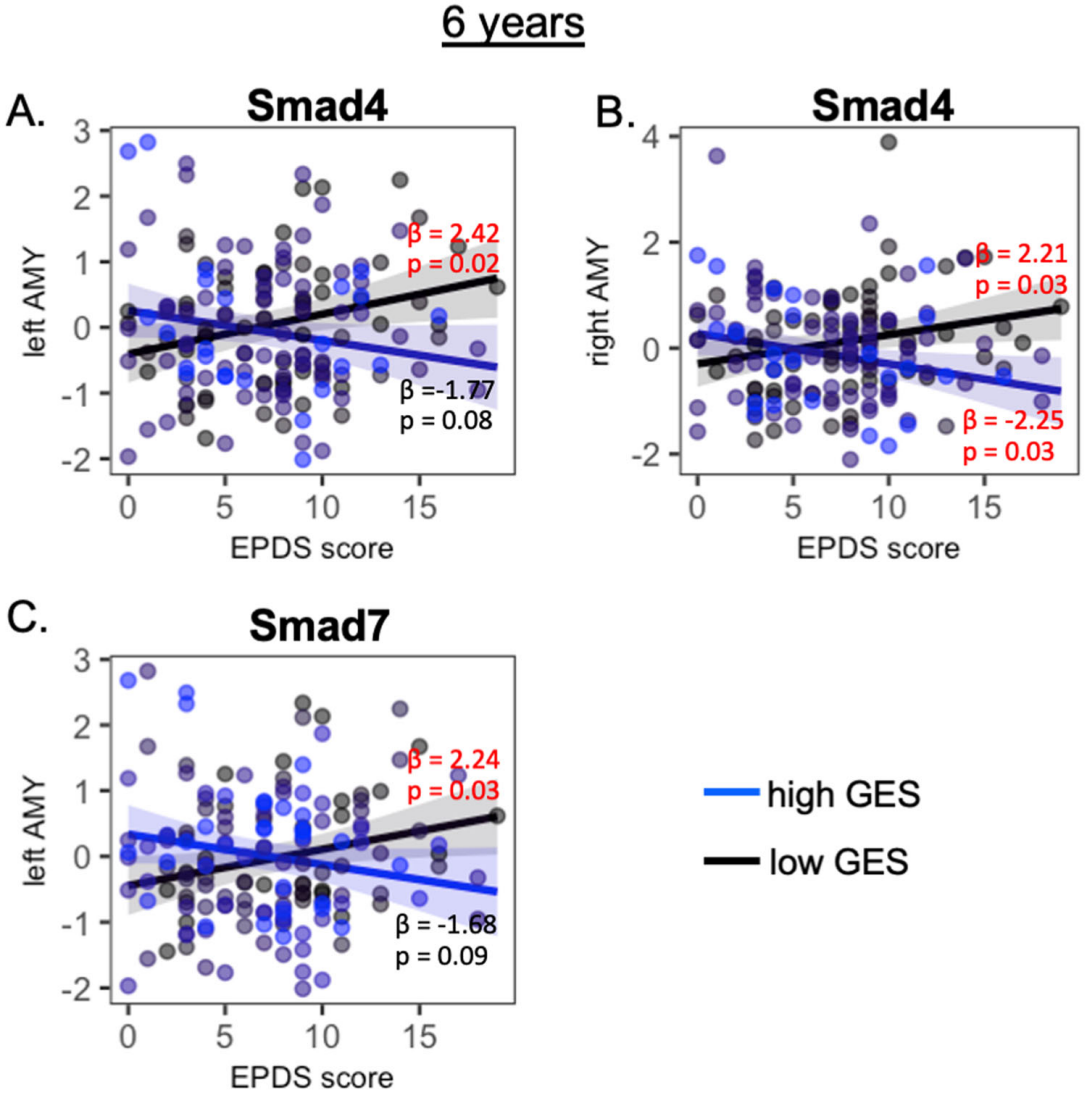
Interaction between prenatal maternal depression and Smad on maygdala volumes. Post hoc analysis of the interaction between prenatal maternal depression and the genetic variants of Smad4 (**A**, **B**) and Smad7 (**C**) on the amygdala volume at the age of 6 years. The black and blue lines/dots represent the groups with low and high genetic expression scores (GES), respectively. *β* and *p* values in red indicate statistical significance.

Smad4 and Smad7 variants also interacted with prenatal maternal depressive symptoms on the bilateral amygdala volumes at the age of 6 years (Table 2). Post hoc analysis revealed that, in 6-year-old children with a low GES of Smad4 and Smad7, greater prenatal maternal depressive symptoms predicted a larger left amygdala volume (Smad4: *p* = 0.02, Fig. 4A**;** Smad7: *p* = 0.03, Fig. 4C). Among 6-year-old children with a low GES of Smad4, higher levels of prenatal maternal depressive symptoms were related to a larger right amygdala volume (*p* = 0.03, Fig. 4B), while among 6-year-old children with a high GES of Smad4, higher levels of prenatal maternal depressive symptoms were related to a larger right amygdala volume (*p* = 0.03, Fig. 4B).

The above interaction effects remained the same even after additionally controlling for the same genetic variation of mothers (see Table S1 in Supplementary Material), suggesting that our findings on the amygdala was independent of mothers’ genetic variants. Moreover, we considered the primary visual cortex as a reference region and ran the MixGE analysis on the thickness of the primary visual cortex. No statistical significance was revealed on the interaction between prenatal maternal depressive symptoms and the genes shown in Fig. 1 on the visual cortical thickness (all *p* > 0.05). Together with the amygdala finding, this result suggested the potential modulation of the canonical TGF signaling pathway specific to emotion-related regions in the context of prenatal maternal depressive symptoms.

## Discussion

This study investigated the modulatory role of the canonical TGF-β signaling pathway in linking pregnancy depression and child brain development. Our findings were best interpreted for a general population. Our previous studies using the same sample showed the impacts of prenatal maternal depressive symptoms on the amygdala volume and microstructure^7,9^. In this study, we additionally showed that the functional genetic variants of TGF-βRI modulated the association between prenatal maternal depressive symptoms and the amygdala volume consistently from birth to 6 years of age despite a trend of significance at 4.5 years of age. Children with a lower GES indicative of reduced TGF-βRI expression exhibited larger amygdala volumes in relation to greater prenatal maternal depressive symptoms. Moreover, children with a lower GES of TGF-βRII, Smad4, and Smad7 showed larger amygdala volumes at 6 years of age in relation to greater prenatal maternal depressive symptoms. These findings support the idea of the canonical TGF-β signaling pathway as one of the possible biological pathways acting to modulate the influence of maternal depressive symptoms in pregnancy on child neurodevelopment.

The canonical TGF-β signaling pathway is known to play a multifunctional role in the regulation of embryonic development, immunity, and cellular functions, such as cell proliferation, differentiation, and apoptosis^44–46^. In this study, children with the genetic variants of TGF-βRI, TGF-βRII, and Smad indicative of their respective reduced expression exhibited larger amygdala volumes when exposed to greater maternal depressive symptoms in utero. When introducing in vitro exposures to high concentrations of cortisol that mimics depression, TGF-β-Smad signaling is downregulated in neurons^47^. TGF-βRI and Smad4 mutations are found in patients with neurodevelopmental disorders and their expression disrupts neuronal morphogenesis in both mouse and human neurons^48^. In patients with MDD, the TGF-β serum levels are reduced^27,28^, associate with depression severity^29^, and are increased after treatment with fluoxetine, venlafaxine, or paroxetine^49^. Children high with maternal depressive symptoms during pregnancy show a large amygdala volume^7,11^. Together with these findings, this study provided new insight into the canonical TGF-β signaling pathway in modulating the amygdala development in children who are exposed to maternal depression in utero.

This study suggests an age-dependent modulation of neurodevelopment through the canonical TGF-β signaling pathway. At the age of 6 years, both TGF-β receptors and Smad modulated the relationship between prenatal maternal depressive symptoms and the amygdala volumes, suggesting the involvement of the canonical TGF-β signaling pathway in the brain development in the context of in utero maternal environment. In contrast, only the genetic variants of the TGF-β receptors influenced the amygdala volume of neonates as a function of maternal depressive symptoms during pregnancy. We did not observe the involvement of the receptor-activated R-Smads, I-Smads, and common Smads at birth. This may imply alternative TGF-β receptor biological pathways during fetal neurodevelopment. TGF-β signaling activates Smad-independent pathways, such as phosphoinositide-3 kinase/AKT and mitogen-activated protein kinase pathways, and neurotransmitter pathways^50^. From the same sample, we previously showed that neurotransmitter/neurotrophic signaling, SNARE complex, and glutamate receptor activity as biological processes underlie influences of high prenatal maternal depressive symptoms on the larger neonatal amygdala volume^51^. These findings suggest that candidate biological mechanisms involve a range of brain region-specific signaling pathways that may converge on common processes of synaptic and inflammatory development.

While this study was preliminary, the neuroimaging datasets was unique in their timing of acquisition. It is nevertheless a modest sample size for genomic analyses. Even though this study by design was a longitudinal study, imaging children in the first 6 years of life was challenging and the overlapping sample among the three time points (*n* = 24) was limited. Hence, this study only investigated whether the interactive effects of the TGF-β genetic factors and pregnancy depressive symptoms on brain development occur at a specific age. Moreover, there is a lack of a replication sample. Our study was best considered as exploratory. However, the genetic variation of TGF-βRI consistently showed the interactive effects on the amygdala volumes across the three time points and multiple genes in the canonical TGF-β signaling pathway acted on the amygdala volumes at 6 years of age. Hence, these findings are of some interest, as the canonical TGF-β signaling pathway has been implicated in depression pathogenesis^34,52,53^. Last but not least, our study did not include potential effects of early-life maternal exposure to adversity or partner support on the brain development of the offspring.

In conclusion, our findings suggest an age-dependent modulation of neurodevelopment through the canonical TGF-β signaling pathway in the context of in utero maternal environment. The canonical TGF-β signaling pathway might potentially serve as targets for the prevention or treatment of maternal mental health during pregnancy, which may optimize the brain development of the offspring.

## Supplementary information

Supplementary Material
