# Supplementary material for: Canonical TGF-β signaling regulates the relationship between prenatal maternal depression and amygdala development in early life
Source: Transl Psychiatry. 2021 Mar 15;11:170. doi: 10.1038/s41398-021-01292-z (PMC7961018; doi:10.1038/s41398-021-01292-z)
Supplement: Supplementary file 1 — Supplementary Material [file 41398_2021_1292_MOESM1_ESM.docx]

**Table S1.** Statistical p-values for the interactions between the genetic variants and prenatal maternal depressive symptoms on the amygdala volumes at birth, age of 4.5 and 6 years when the same genetic variation of mothers was entered as an additional covariate.

| **Gene** | **birth** | | **4.5 years** | | **6 years** | |
| --- | --- | --- | --- | --- | --- | --- |
|  | **left** | **right** | **left** | **right** | **left** | **right** |
| **Ligands** | | | | | | |
| **TGFβ1** | 0.429 | 0.441 | 0.704 | 0.936 | 0.694 | 0.976 |
| **TGFβ2** | 0.598 | 0.305 | 0.233 | 0.138 | 0.725 | 0.752 |
| **TGFβ3** | 0.516 | 0.347 | 0.175 | 0.274 | 0.203 | 0.506 |
| **Type I and II receptors** | | | | | | |
| **TGFβ-RI** | 0.166 | 0.005 | 0.059 | 0.048 | 0.045 | 0.047 |
| **TGFβ-RII** | 0.384 | 0.718 | 0.629 | 0.821 | 0.042 | 0.345 |
| **TGFβ-RIII** | 0.648 | 0.181 | 0.760 | 0.552 | 0.014 | 0.512 |
| **R-Smads** | | | | | | |
| **Smad2** | 0.472 | 0.112 | 0.486 | 0.442 | 0.020 | 0.334 |
| **Smad3** | 0.152 | 0.382 | 0.434 | 0.439 | 0.295 | 0.904 |
| **Co-Smads** | | | | | | |
| **Smad4** | 0.752 | 0.080 | 0.721 | 0.181 | 0.025 | 0.019 |
| **I-Smads** | | | | | | |
| **Smad7** | 0.167 | 0.148 | 0.743 | 0.623 | 0.032 | 0.538 |

red: uncorrected p<0.05; uncorrected blue: p<0.1.


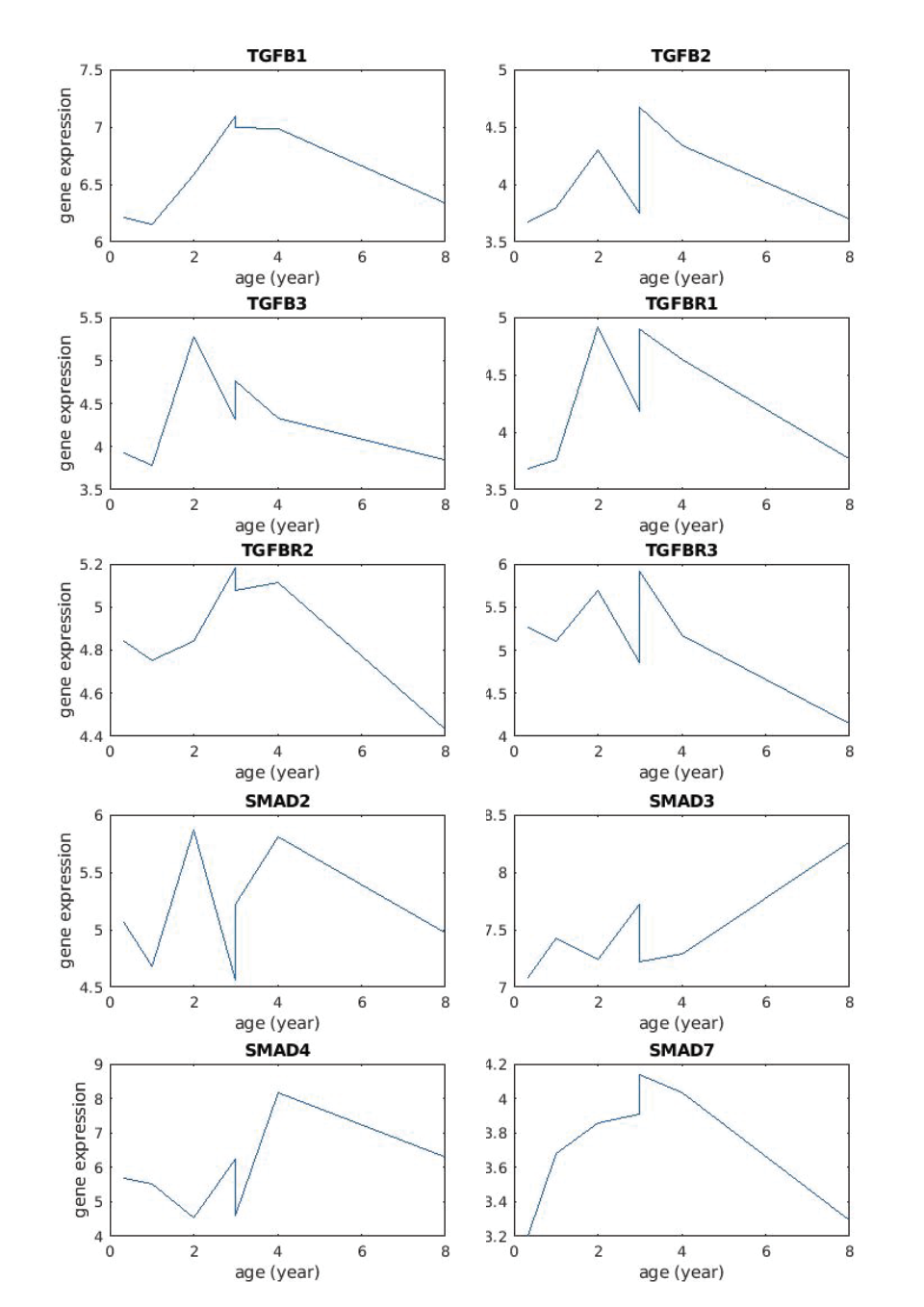


**Figure S1.** The expression level of the genes in the canonical TGF-β signaling pathway in the first eight years of life. The gene expression levels were extracted from the BrainSpan sample (<https://www.brainspan.org/static/download.html>).
